# Attitudes to and management of fertility among primary health care physicians in Turkey: An epidemiological study

**DOI:** 10.1186/1471-2458-5-33

**Published:** 2005-04-05

**Authors:** Hikmet Hassa, Unal Ayranci, Ilhami Unluoglu, Selma Metintas, Alaeddin Unsal

**Affiliations:** 1 Medical Faculty, Gyneocology Department, Osmangazi University, 26480 Meselik-Eskisehir, Turkey; 2 Medico-Social Center, Osmangazi University, 26480 Meselik-Eskisehir, Turkey; 3 Medical Faculty, Family Medicine Department, Osmangazi University, 26480 Meselik-Eskisehir, Turkey; 4 Medical Faculty, Public Health Department, Osmangazi University, 26480 Meselik-Eskisehir, Turkey

## Abstract

**Background:**

The subject of infertility has taken its place in the health sector at the top level. Since primary health care services are insufficient, most people, especially women, keep on suffering from it all over the world, namely in underdeveloped or developing countries.

The aim of this study was to determine primary care physicians' opinions about the approach to infertility cases and their place within primary health care services (PHCSs).

**Methods:**

The study was conducted between October 2003 and April 2004. The study group comprised 748 physicians working in PHCSs. They were asked to fill in a questionnaire with questions pertaining to infertility support, laboratory and treatment algorithms, as well as the demographic characteristics. The data was evaluated using the chi square test, percentage rates and a logistic regression model.

**Results:**

The multivariate analyses showed that having a previous interest in infertility and having worked for a postgraduate period of between 5–9 years and ≥10 years were the variables that most positively influenced them in their approach to cases of infertility (p < 0.05, each one).

Just 28.7% of the physicians indicated that they believed cases of infertility could be evaluated at the primary care level. The most frequently proposed reason for indicating 'difficulty in practice' (n = 533) was inadequate provision of equipment in PHCSs (55.7%). The physicians reported that they were able to perform most of the supportive treatments and proposals (between 64.6%–87.7%). The most requested laboratory investigations were the instruction of patients in taking basal body temperatures and semen analysis (89.7% and 88.7%, respectively). The most preferential course of treatment was that of sexually transmitted diseases (95.5%).

**Conclusion:**

It is clear that not enough importance is attached to the provision of care to infertile couples within PHCSs. This leads us to conclude that an integration of infertility services in primary care would be appropriate after strengthening the PHCSs.

## Background

Infertility affects between 8–20% of couples around the world at some stage during their reproductive years. As the figures suggest, it has become a common health and communal problem in terms of not only the couples involved, but also their health team and the social environment [1-3].

The level and patterns of infertility apparently vary widely, being found less in developed countries, and more in underdeveloped or developing countries [4].

Although the damage that infertility causes to health is generally emotional, it can also have a negative effect on an individual's health, quality of life, and life expectancy [5]. Thus, social and psychological support as well as medical diagnosis and treatment is of great importance while treating couples with infertility [6].

The likelihood of a couple presenting to their primary health care physicians (PHCPs) for the first time with fertility concerns, i.e. wanting to conceive a child but having difficulty, at some point during their reproductive lives is high [7]. PHCPs must decide on the circumstances under which these patients should be referred to specialist reproductive health centers [8,9]. Even when PHCPs refer these cases to specialists, they are still expected to provide counseling and information for patients in every aspect of Assisted Reproductive Technologies, such as in vitro fertilization and intracytoplasmic techniques [10]. This places PHCPs at the heart of all issues relating to infertility [3,6,11,12].

The 21st World Health Assembly (1968) expressed the opinion that every family should have the opportunity of obtaining information and advice on problems connected with family planning, including fertility and infertility, and suggested the integration of these services within primary health care services (PHCSs) [13]. Similarly, in the Alma Ata Declaration of 1978, emphasis was placed on the World Health Organization's role in strengthening PHCSs and the correlation of infertility services with primary health care (PHC) [14].

As with other types of disease, not enough importance has been given to the provision of infertility services by PHC in Turkey, a state reflected in most developing countries [15]. Since Turkey lacks a PHC physician system, it has yet to form a standard in other disciplines, as well as in its approach to cases of infertility. However, in developed countries, PHCPs are in a unique position to provide patient education, offer ongoing counseling, and give psychological help to couples who experience problems with fertility [16].

The purpose of this study was therefore to contribute to the formation of a model for the integration of infertility services within PHCSs, and to both determine PHCPs' opinions about the approach to infertility cases and their place within PHCSs.

## Methods

### Sampling and subjects

This study was carried out in 7 cities, namely Eskisehir, Konya, Bilecik, Bolu, Duzce, Kutahya and Afyon, selected by a random sampling method from 18 cities in Central Anatolia, Turkey during 6 months between October 2003 and April 2004. 1,212 PHCPs were working in 110 PHCSs in the seven cities during the study. PHCPs were recruited randomly during routine visits to local health institutions. The sample capacity to constitute the study was estimated as 384 physicians, assuming that the occurring frequency of infertility was 50%. In the study, since the cluster sampling method was used as the sampling method, the sample volume was determined as 768, multiplying by two the number of 384. These physicians were then further selected by a random sampling method as proportional with the number of the physicians in those cities. All 748 of the physicians contacted agreed to fill in the questionnaires (100%). A total of 20 physicians could not participate in the study due to the taking leave or being absent at the time of the study.

### The interview schedules

The dates on which the study would be conducted were determined in cooperation with government health officials in the cities concerned. The PHCSs were visited with an official of health authority. The physicians completed questionnaires in the presence of a member of the research team, with the researchers being on hand to explain any questions that the physicians found incomprehensible.

### Development of the questionnaires

In the first part of the questionnaire, the physicians were asked to state their demographic characteristics. The second part of the questionnaire, which was prepared with the benefit of reference to previous studies [17-20] and according to infertility treatment algorithms, included three phases. In the first phase, PHCPs were asked to reply to questions concerning to circumstances of providing supportive treatment and proposals to infertile individuals; in the second phase, which laboratory support was requested; and in the third phase, the things to be done during treatment.

### Data analysis

The data were evaluated by SPSS with the chi square (χ^2^) test and percentage (%) ratios where possible. The evaluation of primary care infertile cases by PHCPs was accepted as the dependent variable. Independent variables, which can affect the dependent variable, were determined using a logistic regression model, which is used as a multivariate analysis method. The variables which produced significance of p < 0.05 in the univariable analyse were applied to the model. The physician's age, sex, working time, and the status of having previously evaluated a case of infertility were chosen as independent variables.

### Informed consent for the study

Local authorities, such as the Osmangazi University School of Medicine and the health authorities in the cities concerned, approved this study. All physicians gave their informed consent prior to their inclusion in the study.

## Results

The average age of the physicians included was 33.4 ± 5.5. The rates of the male and female PHCPs were 52.1% (390) and 47.9% (358), respectively. The average working period of the physicians was 8.6 ± 5.2 years.

When the physicians were asked about the proportion of applications to receive advice for an infertility concern from within the total number of applications, 124 (16.6%) responded 0%; 491 (65.6%) for 1–3%; 95 (12.7%) for 4–5%; and 38 (5.1%) for 6% and over.

Just 28.7% of the physicians indicated that infertile cases could be evaluated at PHC. The most frequently stated reason given with an indication of 'practice difficulty' (n = 533) was a lack of devices/equipment at PHCSs (55.7%). Detailed data on the approach to patients with infertility problems is shown in Table 1.

**Table 1:** The physicians' attitudes about evaluation of infertile cases at primary care and the reasons that were put forward by those who indicated that there was practice difficulty

**Attitudes**	**n = 748(100%)†**	**95% CI**
Those believing that infertile cases could be evaluated at primary care	215 (28.7)	25.7–31.7
Those believing that infertile cases could be evaluated at primary care but that the application would prove difficult	76 (10.2)	8.0–12.4
Those believing that infertile cases cannot be evaluated at primary care level	457 (61.1)	59.3–62.9

Reasons proposed by those indicating that there was difficulty in application*	**n = 533(100%)†**	**95% CI**

Supply of logistics is inadequate (lack of device and equipment at primary care)	297 (55.7)	51.5–59.9
Only specialists should evaluate	179 (33.6)	31.6–35.6
My level of education in this field is insufficient	149 (30.0)	26.1–33.9
There is not enough time at primary care	46 (8.6)	6.2–11.0
It is a loss of time for patient	43 (8.1)	5.8–10.4

†Since more than one reason is proposed, the proportion exceeds 100%. * Those who believe that while infertile cases could be evaluated at primary care the practice could prove difficult (n = 76) and those who believe that the practice would not be possible at primary care (n = 457)

Table 2 shows the opinions of PHCPs related to the evaluation of cases with infertility. With the exception of items 11., 12. and 13. (p > 0.05, each one), more physicians indicated that PHC was an appropriate place for evaluation than did those who did not. These majorities were significant in 6 of the 7 items (items 1., 3., 4., 5., 6. and 7.) related to support, treatment and proposals. However, in the group related to the use of laboratory investigation, only four of the twelve items showed a significance (items 8., 9., 14., and 16.). The opinions of the physicians on treatment were significant for all the items (p < 0.05, each one).

**Table 2:** Primary health care physicians' opinions related to evaluation of infertile cases at primary care

**Physicians' opinions concerning supportive treatment and proposals for infertile couples**	**Physicians' opinions related to evaluation of infertile cases at primary care**		Total	
			
	Appropriate* n = 291(38.9%)	Inappropriate** n = 457(61.1%)	n(%) 748(100)	p
1.I could administer rubella prophylaxis	209(72.2)	295(64.6)	504(67.4)	**0.030**
2.I could begin folic acid support	262(90.0)	401(87.7)	663(88.6)	0.336
3.I can encourage couples to avoid cigarettes, alcohol and drug abuse	258(88.7)	376(82.3)	634(84.8)	**0.018**
4.I can resolve obesity problems	251(86.3)	348(76.1)	599(80.1)	**0.001**
5.I can prevent testicular hypertermia by advising on appropriate clothing to be worn	266(91.4)	375(82.1)	641(85.7)	**0.000**
6.I can inform of coit order	269(92.4)	381(83.4)	650(86.9)	**0.000**
7.I can investigate the distress that childlessness causes	255(87.6)	349(76.4)	604(80.7)	**0.000**

**Physicians' opinions concerning request for laboratory investigations at primary care level in evaluating infertility in couples**				

8.I have semen analyses done	258(88.7)	368(80.5)	626(83.7)	**0.003**
9.I evaluate one value progesterone hormone for ovulation between the 22. and 24. days of cyclus	217(74.6)	309(67.6)	526(70.3)	**0.042**
10.I request an evaluation of FSH, LH, E2 and Prolactin on the 2. and 4. days of the male's cycle	193(66.3)	292(63.9)	485(64.8)	0.498
11.I perform an ultrasonic folliculometric ovulation follow-up	118(40.5)	204(44.6)	322(43.0)	0.271
12.I diagnoses policystic ovary disease by means of folliculometric measure	117(40.2)	211(46.2)	328(43.9)	0.109
13.I diagnoses uterus anomalies by ultrasound	123(42.3)	226(49.5)	349(46.7)	0.055
14.I teach the patient to measure basal body temperature	261(89.7)	373(81.6)	634(84.8)	**0.003**
15.I investigate thyroid functions if the result of a physical exam is positive	241(82.8)	363(79.4)	604(80.7)	0.252
16.I investigate prolactine levels if there is a history of galactore history or if a physical exam is positive	238(81.8)	346(75.7)	584(78.1)	**0.050**
17.I have patient's adrenal hormones investigated if the results of hirsutismus or a physical exam are positive	226(77.7)	331(72.4)	557(74.5)	0.109
18.I evaluate vaginal or urethral discharge by microscope in cases with complaint	194(66.7)	284(62.1)	478(63.9)	0.209
19.I ask whether or not hysterosalpingographic study has previously been conducted and if tubes were open	194(66.7)	300(65.6)	494(66.0)	0.774

**Physicians' opinions about treatment of infertile cases**				

20. I can treat sexually transmitted diseases	278(95.5)	412(90.2)	690(92.2)	**0.007**
21.I can guide patients I have diagnosed as infertile to a higher healthcare level, and can correlate a follow- up treatment for patients with that center	270(92.8)	388(84.9)	658(88.0)	**0.001**
22.I can administer clomiphen citrate treatment for ovulation	107(36.8)	136(29.8)	243(32.5)	**0.046**
23.I can administer bromocriptin in cases with hyperprolactinemia	123(42.3)	142(31.1)	265(35.4)	**0.002**
24.I can perform the treatment of hyperandrogenemia	92(31.6)	103(22.5)	195(36.6)	**0.006**
25.I can administer metphormine derived drugs for cases with policystic ovary disease	111(38.1)	137(30.0)	248(33.2)	**0.021**
26. I can administer gonodotrophinler for ovulation	94(32.3)	113(24.7)	207(27.7)	**0.024**
27.I can perform hormonal treatment for male infertility	94(32.3)	102(22.3)	196(26.2)	**0.002**
28. I can perform insemination with a males split ejaculation	59(20.3)	56(12.3)	115(15.4)	**0.003**

*Those who believed that infertile cases could be evaluated at primary care (n = 215) and Those who believed that while infertile cases could be evaluated at primary care the practice could prove difficult (n = 76), ** Those who think that infertile cases could not be evaluated at primary care (n = 457)

The result model of the logistic regression analyse is presented in Table 3. 40.2% (164/408) of the physicians working in cities with infertility centers, and 37.4% (127/340) of those who did not, reported that they saw appropriate the evaluation of infertile couples (p = 0.427). This proportion was reported at 45.4% (49/108) for physicians who had received education related to reproductive health after graduating, whereas the figure was 37.8% (242/640) for those who had received no education (p = 0.136); 36.2% (71/196) for physicians having graduated from medical faculties with have infertility centers, but 39.9% (220/552) for those who had not (p = 0.370) (Unshown data).

**Table 3:** Multivariate analysis results for the relationship between appropriate and inappropriate behaviour to infertile cases according to the demographic characteristics of the primary health care physicians

Demographics	**Those seen as appropriate n = 291(38.9%)**	**Those seen as inappropriate n = 457(61.1%)**	**Total n = 748 (100%)**	**Odds Ratio (95% Confidence Interval)**	**p**
*Sex*					
Female	123(34.4)	235(65.6)	358(47.9)	1	
Male	168(43.1)	222(56.9)	390(52.1)	1.27 (0.93–1.74)	0.126
*Age groups*					
Age 24–29 years	63(30.3)	145(69.7)	208(27.8)	1	
Age 30–39 years	183(40.4)	270(59.6)	453(60.6)	0.784 (0.473–1.30)	0.345
Age ≥40 years	45(51.7)	42(48.3)	87(11.6)	0.974 (0.475–1.99)	0.942
*Post graduation period*					
1–4 years	43(25.3)	127(74.7)	170(22.7)	1	
5–9 years	107(38.1)	174(61.9)	281(37.6)	1.785 (1.04–3.05)	**0.034**
≥10 years	141(47.5)	156(52.5)	297(39.7)	2.418 (1.298–4.502)	**0.005**
*Previously dealing with the management of an infertile couple*					
No	123(35.6)	177(64.4)	345(46.1)	1	
Yes	168(41.7)	280(58.3)	403(53.9)	1.870 (1.368–2.554)	**0.034**

The independent variable most influencing the physicians' opinions on this subject was having previously dealt with such a case during their occupational career (p < 0.05) and having postgraduate experience in the field of PHC for a period of 5–9 years and ≥10 years (p < 0.05, each one).

46.1% (345/748) of the PHCPs reported that they had been involved in the evaluation/treatment of an infertile couple or couples. While 4.3% (15/345) of the physicians reported having had no involvement in the evaluation of infertile couples, the data provided by the majority of the physicians (95.7%, 330/345) for the procedures most frequently administered to patients are given in table 4. According to this data, more than half of the procedures consisted of the referral of patients to a higher tier health center (57.6%), the provision of information and counseling (58.5%), followed by requesting that laboratory and radiological investigations be done (27.9%).

**Table 4:** The practices of physicians who had been involved in provision of services to infertile cases until the time of the study

	Total n = 330(100%)*
Provision of information and counseling (concerning iron deficiency, menstruation disorders, folic acid deficiency, ovulation periods, alcohol-cigarette-coffee drinking, coit order, behaviour pertaining to raising sperm quality, basal body temperature)	193(58.5)
Reference to a secondary healthcare center	190(57.6)
Request laboratory and radiological investigations (PRL, E_2_, FSH, LH, Thyroid tests, USG, Sperm analysis) in order to diagnose the reason for infertility (Galactorhea, hirsutism, polycystic ovary syndrome)	92(27.9)
Treatment of sexually transmitted diseases	36(10.9)
Psychological support to decrease the distress of infertile couple	35(10.6)
Case history and physical examination	28 (8.5)
Hormonal treatment for infertility (gonadotropine treatment, drug treatment for ovulation, bromocriptine treatment, polycystic ovary syndrome treatment, hyperandrogen treatment)	17 (5.2)
Performance of a follow-up for patients returning to primary care after referral to a higher health center	15 (4.5)

*Since the physicians indicated that they had performed more than one treatment, the total proportion exceeds 100%.

6.9% (23/330) of the physicians indicated that they had not experienced any difficulties during the approach to their patients. The various difficulties of 307 (93.1%) of the physicians stating having experienced a problem during consultation are shown in Table 5. According to the information provided, the most widely reported difficulty physicians had experienced was an inadequacy of logistics (35.2%), followed by feelings of despondency on the patient's part (32.2%).

**Table 5:** Difficulties physicians had experienced during approach

	n = 307(100%)*
Inadequate supply of logistics (lack of device-equipment-staff)	108(35.2)
Patient's lack of harmony (psychological problems, illogical behaviour, couples not attending together, low cultural level of couples, couples remained unconvinced)	99(32.2)
Management mistakes/errors (the absence of communication between primary health care and hospitals, the absence of a comfortable atmosphere in which patients with infertility can speak)	72(23.5)
Physician's lack of education/knowledge-physician's lack of post-graduate education	71(23.1)
Patients' not having health insurance, money problems of patients	27 (8.8)
Lack of belief and confidence in the primary health care physician	17 (5.5)

*Since more than one reason is proposed, the proportion exceeds 100%.

Table 6 reports suggestions forwarded by PHCPs' for the provision of better service to infertile couples. The physicians most commonly recommended the encompassment of infertility matters in family planning services, as well as the introduction of an on-going postgraduate education programme (73.1% and 70.1%, respectively).

**Table 6:** Proposals forwarded by primary health care physicians for the provision of better service to infertile cases

**Practices**	n = 748(100%)*
Inclusion of the subject of infertility in Family planning services	547(73.1)
A planned post-graduate continuing education programme	524(70.1)
A strengthening of routes of communication between primary care and other care services	520(69.5)
Improvement of laboratory conditions	503(67.2)
More support for this subject in graduation education	398(53.2)

*Since more than one reason is proposed, the proportion exceeds 100%.

## Discussion

This study is the first large scale Turkish epidemiological study conducted to learn PHCP's management of infertile patient, their attitudes to fertility treatment, and their recommendations for the provision of better service in the future.

In our study, a near absence of awareness on the part of doctors that nearly 1/5 (16.6%) of all patients entering PHC applied for consultation for fertility problems, and that about 2/3 (65.6%) of the same physicians reported that they believed that the issue of infertility did not have a place during routine examination is a cause for concern.

In this study, the proportion of the physicians who viewed the evaluation of infertile cases as appropriate in PHCSs was 38.9%. This proportion was reported at around 27.0% in a study by Ittner et al (2000) [21]. That the proportions are less than 50% shows that the physicians in different countries have similar thoughts about the evaluation of infertility in PHC.

In the current study, most physicians reported that they could perform a large part (67.4%-88.6%) of the necessary supportive treatments and consultations directed at fertility in the preconceptional period. In parallel to this finding, those physicians who had been involved in cases of infertility during their working life stated that they had performed 82.4% of supportive treatment and recommendations, including offering psychological support (10.9%), the taking of patient histories and physical examinations (8.5%), and the following- up of patients returning after having been referred to higher-level healthcare centers (4.5%) (Table 4). These results reveal a balance between what the physicians reported they did and what they would do. In the study by Heyes et al (2004) [22], a proportion of between 79.1% and 86.7% of PHCPs indicated the practicality of performing support treatment and recommendations and that the most suitable location was within PHC, whereas the proportion of physicians stating that a hospital setting was the most appropriate place for the performance of these duties was only 1.2%.

In this study, the physicians reported that the area of supportive treatment and recommendations for treatment they least preferred was concerning rubella prophylaxis (67.4%). By means of a suggestion for this low proportion, we can offer the explanation that rubella vaccination in Turkey is not included in routine vaccination schema, and that, therefore, physicians do not have much knowledge of this vaccination. However, Turkish studies on cord blood indicate that 15% of those who will become mothers are sensitive to rubella [23]. This demonstrates the need for fortification of knowledge on rubella prophylaxis at the level of the physicians.

In this study, a great majority of physicians reported that in terms of the provision of laboratory support they were able to perform an investigation of semen analyses and of a one-value progesterone hormone for ovulation (83.7% and 70.3%, respectively). However, areas that they felt less confident in were performing such investigations as folliculometric ovulation follow-up by ultrasound, the diagnosis of polycystic ovarian disease by means of folliculometric measurement, and the diagnosis by ultrasound of uterine anomalies (between 43% and 46.7%). These findings are consistent with those of other studies on distribution of duties performed by PHCPs [5,11,15].

In this study, the opinions of both sets of physicians on the evaluation of cases of infertility in PHCSs were positive as expected. The proportions of the physicians responding that they were able to perform treatment of sexually transmitted diseases and referral and follow-up of patients diagnosed as infertile were rather high (92.2% and 88.0%, respectively), whereas those reporting being able to perform more heavy treatments, regarded as secondary care duties, such as ovulation induction was rather low (between 36.6% and 15.4%). These proportions show that PHCPs working in the region have sufficient reproductive health knowledge and that when physicians are presented with appropriate conditions they are able to perform follow-up to and treatment of infertility.

The proportion of physicians in this study reporting that they could evaluate vaginal or urethral discharge through microscopic investigation was less than that of physicians able to offer treatment for sexually transmitted diseases (63.9% and 92.2%, respectively). This result is important from the viewpoint that the approach offered to patients by physicians in the region is to directly use empirical treatment without performing detailed investigations, and that there was a big gap in adequacy of laboratory equipment available in PHCSs.

In this survey, it was determined that approximately two thirds of the physicians were not comfortable performing procedures usually offered in secondary care, such as performing insemination with a split ejaculation (items 22. to 28.). In line with our study results, some guides [24] suggested that if criteria related to secondary care, such as those mentioned above were present, such patients should be referred to higher-level health centers.

In our study, a rather low proportion of physicians reported that they were able to administer clomiphen citrate and bromocriptin (32.5% and 35.4%, respectively) to their patients. In some studies [25], although emphasizing that these medicines could be administered at PHC level, the fact that this approach is rather new and not approved all over the world, may indicate why the physicians in our study may have marked these alternatives less.

Physicians reported that having a previous interest in infertility and having worked for a longer postgraduate period were the important independent variables that most positively influenced them in their approach to cases of infertility. This finding shows that, as the time passed after graduation and the number of infertility cases dealt with increases, so do the physicians' levels of confidence.

In this survey, more than half of the physicians who had been involved in cases of infertility (57.6%) reported that they directly referred infertile patients to a higher-level healthcare provider. The result of a study conducted in Germany is in line with our study result, were the proportion was reported as 65% [21]. In the same study [21], it was found that 43% of the physicians did not know of any patient in their practice who had had fertility problems. In a similar study [26], a majority of the physicians placed infertility within the realm of fertility specialists.

In the present study, the most frequent reason seen for physicians reporting the referral of infertile patients was a lack of logistics in the PHCSs (35.2%, 108/307). This reason, taking into consideration the health care system in our country, reveals that even when physicians want to perform treatment of patients in the primary healthcare system, the system is unable to deal with the demand. In a study by Heyes et al (2004) [22], the most reported reason for experiencing difficulties while evaluating cases at primary care level was due to service access problems and resource constraints

In our study, the second most frequently reported reason was the patient's lack of harmony (32.2%). This finding is consistent with other study results [21,26-28]. The study by Ittner et al (2000), [21] in particular reported that almost the same proportion of physicians emphasized the influence of fertility problems on personality, sexuality, social acceptance, and mental health status.

A great proportion of the difficulties that physicians experienced during their approach arose from reasons not directly connected to the patient. If lack of knowledge on the part of the physician, inadequacy in the supply of logistics, and managerial errors were to be removed, an increase in information being given to the patient and a 32.2% decrease in patients experiencing feelings of despondency may be anticipated.

Of the difficulties that physicians reported having encountered during consultation, a further important reason that emerged was lack of communication between health institutions (23.5%). In our country, since there is still no coordination between health institutions, this absence negatively affects the follow-up of patients with infertility. If higher tier health centers were to inform PHCSs of the treatment administered, PHCPs would have an increase in self-confidence.

In our study, three-quarters of all the PHCPs while reporting in the recommendations section mentioned the importance of education both before graduation and post graduation. In a similar way to the reports of other studies [22,29], the emphasis was placed on providing across the board training to all primary health care workers in order to enable them to deliver this care more confidently and raise awareness and skills.

## Conclusion

The concern held by many that cases are not given due care and attention, particularly care offered to couples with infertility, within PHCSs [26-30], also appeared in this study. Our conclusion, based on the recommendations provided by physicians working in PHC, was that the implementation of an education programme, thus boosting self-confidence, would have the greatest impact on changing the attitudes of physicians towards cases of infertility.

Since we have not yet been able to find any study conducted on this subject in Turkey, we recommend that further comprehensive research is needed in order to expose PHCPs' thoughts and attitudes towards infertility.

## Competing interests

The author(s) declare that they have no competing interests.

## Authors' contributions

HH conceived of the study, AU participated in its design and coordination, sequence alignment, collected the data and drafted the manuscript, UI participated in its coordination and collected the data, MS participated in the design of the study, performed the statistical analyses and collected the data, UA participated in its coordination. All authors read and approved the final manuscript.

## Pre-publication history

The pre-publication history for this paper can be accessed here:


